# Cultures of engagement: The organizational foundations of advancing health in immigrant and low-income communities of color

**DOI:** 10.1016/j.socscimed.2016.02.003

**Published:** 2016-09

**Authors:** Irene Bloemraad, Veronica Terriquez

**Affiliations:** a Department of Sociology, University of California, Berkeley, United States; b Department of Sociology, University of California, Santa Cruz, United States

**Keywords:** United States, Community-based organizations, Collective action, Civic engagement, Youth, Immigrant communities, Low-income minorities, Cultures of health

## Abstract

A rich civic infrastructure of community-based organizations (CBOs) can help generate, diffuse and maintain a culture of engagement and health that benefits marginalized populations most at risk for illness, disability, and poor health. Attention to CBOs advances “meso-level” frameworks for understanding health cultures and outcomes by going beyond attention to social networks and social identities. We focus on three mechanisms: CBOs can (1) empower individuals by developing civic capacity and personal efficacy; (2) foster solidarity by building networks, social identities and a shared commitment to collective well-being; and (3) mobilize people to have a voice in health-related policies and programming, thereby affecting community well-being. We draw on theory and research in sociology, political science and psychology, and we illustrate the utility of a CBO approach by examining survey and semi-structured interview data from participants in youth civic groups in 13 low-income, predominantly immigrant communities in California. Interview data illustrate the ways in which CBOs enhance members' civic capacities, provide a sense of empowerment and efficacy to engage in healthy behaviors, develop solidarity among diverse participants, and elaborate networks among those committed to community well-being. We also discuss CBO-led campaigns in which youth mobilized for change in policies and practices of local institutions to illustrate possible community-wide health consequences of CBO engagement. CBOs can thus generate individual-level well-being effects, and reduce structural barriers to good health through changes in the broader environment.

## Highlights

• Civic groups can function as meso-level determinants of health and well-being.
• Civic groups can empower individuals, build solidarity and facilitate policy change.
• We illustrate with indepth interviews and surveys of minority youth in California.
• We discuss cases of community-based organizations' health-related initiatives.

## 1 Introduction

As defined by the new Robert Wood Johnson Foundation initiative, a culture of health promotes individual and community well-being, creates physical and social environments that prioritize health, and supports equitable healthy living for everyone (Plough and Trujillo, 2015, Robert Wood Johnson Foundation, 2014). The goal of equitable health faces challenges, however, in societies such as the United States where life chances are stratified by income, education, racial minority status and, for some immigrants, by legal status. We can place individuals' health within larger social, economic, cultural and political environments, but doing so can make ameliorating health outcomes a daunting enterprise. How can ordinary people promote a culture of health, improving health in their communities and their own lives, in the face of policy and macro-structural constraints?

We argue that organized civil society groups form a bridge between individual actions, on the one hand, and changes in policy, cultures, social hierarchies and economic structures, on the other. A rich infrastructure of formalized civic groups can contribute to generating, diffusing and maintaining a thriving “culture of health” among marginalized populations most at risk for illness, disability and poor health. In this article, we advance a conceptual framework for theorizing the role of community-based organizations for a culture of engagement and health and we offer exploratory evidence to illustrate this approach's potential.

We focus on three mechanisms through which community-based organizations (CBOs) can foster a culture of health, building on themes of mobilization and mutuality in this special issue. We argue that CBOs can (1) empower individuals by developing civic capacity and personal efficacy; (2) foster solidarity by building networks, social identities and a shared commitment to collective well-being; and (3) mobilize people to have a voice in health-related policies and programming. A sufficient density and diversity of high-quality civic groups can facilitate healthy responses to economic, social, and political marginalization.

We draw on theory and research in sociology, political science and psychology to build our argument, and we illustrate its utility with original data from youth civic groups in low-income Californian communities. We focus on young people of color, especially immigrants or youth whose parents were born outside the United States. Nationally, approximately one quarter of minors have a foreign-born parent; in California, this figure rises to about one half (Migration Policy Institute, 2015). Immigrant-origin youth from modest socioeconomic origins tend to grow up in racially diverse, low-income communities that have unequal access to the economic, social, and political resources that facilitate health and well-being (Iceland, 2009).

## 2 Theorizing meso-level determinants of health for immigrant-origin and minority populations

Theoretically, we seek to further “meso-level” frameworks for understanding health among low-income, immigrant-origin and minority populations by going beyond a focus on individual or structural determinants of health. At the (micro-) individual level, researchers often investigate biological or behavioral risk factors, from genes that might predispose someone to certain cancers, to activities like smoking or unprotected sex that increase the likelihood of particular health problems. At a (macro-) structural level, studies using social determinants of health or structural vulnerability frameworks examine discrimination, the built environment, inadequate access to care, and other social and economic inequalities to explain why low income, immigrant, and/or racial minority communities are vulnerable to poor health outcomes (World Health Organization, 2008, Quesada et al., 2011). Increasingly, researchers also study the intersection of individual and structural determinants, as in epigenetic studies that examine how poverty influences gene expression, contributing to the likelihood of poor child development (Hertzman and Boyce, 2010).

Researchers have paid less attention to “meso-level” dynamics characterized by small group interaction, or what Gary Alan Fine calls “that space between agentic action and the structural constraints” (2012: 172). In research on health, meso-level analyses have centered on social relations, conceptualized as social networks, social capital or social integration (Berkman et al., 2014, Berkman et al., 2000). Social networks of families, friends, co-workers, neighbors, and other social ties act as a diffusion mechanism of illness and disease. But, they can also influence healthy behaviors, mental health, and well-being (Smith and Christakis, 2008) by providing information, norms and sources of social support. Group-based social identities can also help people cope with, and even recover from, serious stress, illness or trauma (Cruwys et al., 2013, Jetten et al., 2012) and influence motivations for health-promoting behaviors (Oyserman et al., 2014).

Conceptualization of the meso-level as social ties or social identities downplays, however, the institutional and organizational nodes within which networks and group identities form (Shinn and Toohey, 2003), and the ways in which formalized associations shape the usefulness of networks for individuals (Small, 2009), including for health outcomes (Giordano and Lindstrom, 2010, Putnam, 2000). Social networks alone tend to be weak instruments of social change, especially for marginalized populations. In their otherwise excellent synthesis of how social relations link macro-social structures to psychobiological outcomes, Berkman et al. (2000) do not conceptualize a mechanism by which individuals tap networks to collectively modify the macro-social environment. Such collective action is often needed when social, economic and political inequalities structure lives in very different ways.

We thus conceptualize the meso-level to include “civic infrastructures,” which we define as a set of somewhat organized and formalized groups that are neither public institutions nor for-profit businesses. We build on Fine (2012)'s theorization of groups as having some shared history, collective identity, and ongoing social relations in common spaces. These elements are especially important for collective action, which requires the development of a common purpose, solidarity, skills and sense of political efficacy to affect social change (Goodwin and Jasper, 2009). As Shinn and Toohey conclude, reviewing research in psychology and sociology, “Community organizations influence not only people's psychological sense of empowerment but actual power and decision-making in communities” (2003: 445). Recognizing that types of civil society groups can vary cross-nationally or by the population of interest, we posit that a range of civil society groups could generate mechanisms of mobilization and mutuality that we outline below. In California, from where we draw our illustrative cases, non-profit community-based organizations are key elements of local civic infrastructures.

### 2.1 How CBOs matter: mechanisms of mobilization and mutuality

Often, elites engage in health advocacy on behalf of the “unwell” or less privileged at risk for poor health. CBOs, we posit, have the potential to engage marginalized populations directly in cultures of engagement and health by promoting healthy development at the individual level, building collective solidarities, and holding institutions accountable for community well-being. We focus on CBOs for which health care provision is not the primary mission and which facilitate face-to-face encounters around social activities, cultural or religious events, civic engagement, or political mobilization. CBOs can provide physical space to meet, reasons to get together, and assistance with maintaining group interactions over time through regular meetings, training, leadership rotation, and resources. This is especially important for more marginalized and powerless groups. Absent a somewhat formalized organization, mobilization often is limited to sporadic protests or one-off initiatives by a few motivated leaders. Such actions, while effective for registering opposition, have trouble advancing long-term, sustained change.

Consider the grassroots effort facilitated by InnerCity Struggle (ICS), an East Los Angeles CBO we studied. ICS works with youth, parents, and other residents to promote community safety, non-violence, and well-being. Much of its programming focuses on developing civic leadership, providing educational services, and spearheading grassroots advocacy campaigns. Long concerned about poor access to health services, ICS youth members mobilized for approval of the “Wellness Centers Now” resolution, which called for school-based comprehensive health services, including primary and preventative care. With the guidance of adult staff and allies, youth collected over 1000 signed petitions and mobilized peers to attend meetings and hearings held by the Los Angeles Unified School District (LAUSD). On May 13, 2014, LAUSD passed the Wellness Centers Now resolution with a unanimous vote, allocating $50 million for school-based health and wellness centers in high-needs schools.

It is too early to evaluate the health consequences of this victory, but it points to how CBOs can help build skills, generate solidarity and spur mobilization to change health environments. Within public health, development of skills and solidarity, two aspects in capacity building, are key to the community-based participatory model of health interventions (Minkler and Wallerstein, 2008). We recommend going beyond public health interventions to study CBOs of many stripes. We thus conceptualize a ‘culture of health’ approach as requiring active engagement—especially by those most at risk for illness and disease—so that they can enact culturally appropriate policy changes or reduce structural barriers to better health. To theorize how organizations matter, we distinguish effects for participants, and for the broader community.

#### 2.1.1 Psychological empowerment and the development of individual skills

Extensive research documents how CBOs and other voluntary associations can act as ‘schools of democracy,’ teaching participants how to run meetings, speak in public, write letters to officials, organize a protest, and argue issues (Bloemraad, 2006, Verba et al., 1995). While those more likely to participate in civic organizations tend to be people with more education, more privileged positions in society, and employed in more skilled jobs (Verba et al., 1995, Putnam, 2000), organizations that target marginalized or less powerful groups can compensate for structural disadvantages. CBOs can help members understand social issues and build capacity to act on concerns (Ginwright and Cammarota, 2007, Terriquez, 2015), they can provide a place, over a sustained time period, for training, discussion and coordination of activities (Ramakrishnan and Bloemraad, 2008, Terriquez, 2011), and they can promote pride in cultural or minority identity, which furthers empowerment (García Bedolla, 2005, Watts and Flanagan, 2007). Civic engagement, and the psychological empowerment that results, has positive effects on participants' mental health and can reduce the likelihood of violent behavior and substance abuse (Christensen and Person, 2012, Shinn and Toohey, 2003).

Involvement in CBOs can also positively affect members' education and professional development. CBOs that engage young people in grassroots campaigns can enhance participants' academic skills by addressing real world problems, connect them with college-educated role models, refer them to tutoring, or develop professionalism (Kirshner, 2007, Ozer and Wright, 2012, Rogers and Terriquez, 2016). Since educational attainment and occupational status are key predictors of health, CBOs' academic and career-related benefits may translate into better health outcomes in the long-term and promote future civic and political engagement.

#### 2.1.2 Building solidarities around community health

When CBOs engage members in group activities, they can also foster collective identity, solidarity, and collective efficacy (meaning the feeling that individuals can take action together based on mutual trust and connection) (McAdam, 1982, Jetten et al., 2014, Sampson et al., 1997). CBOs can also facilitate coalitions among individuals of different ethno-racial and immigrant backgrounds. Devoting energy to multicultural education and issues that cross racial and immigrant lines can help groups confront internal racial/ethnic tensions (Kurtz, 2002), although being inclusive of diverse constituents does present challenges (Lichterman, 1999, Strolovitch, 2007). When successful, collective efficacy and a common politicized identity can call forth powerful feelings of affective injustice that spur people to action (Polletta and Jasper, 2001, Van Zomeren et al., 2008).

#### 2.1.3 Affecting broader communities through policy and institutional change

Through collective action, CBOs can engage members in advocating for policy and institutional changes that produce positive health externalities for the broader community (Shinn and Toohey, 2003), as with the campaign to place wellness centers in L.A. high schools. Civic organizations can provide a venue to articulate community concerns, identify strategies to address them, and take action to ameliorate them. Research documents cases in which CBOs organize constituents to address asthma management and prevention; homelessness, violence, and obesity prevention; and health-focused land use planning (Minkler and Wallerstein, 2008).

## 3 California youth-serving organizations and cultures of health

How can CBOs promote a culture of engagement and health by empowering individuals, fostering solidarity, and mobilizing people to have a voice in health-related policies and well-being? We offer exploratory evidence for our arguments about the individual and collective effects of participation in CBOs by studying youth-serving organizations in high poverty, predominantly racial minority and immigrant communities. Such communities tend to exhibit comparatively high risk of violence, obesity, heart disease, diabetes, extreme stress, violence, or other negative health outcomes (Dowd et al., 2014, Hertzman and Boyce, 2010). Moreover, lack of access to health care can be acute in communities with many undocumented immigrants (Yoshikawa, 2011). Importantly, a life course health development framework suggests that events and influences during adolescence and young adulthood shape later health status and health behaviors (Mulye et al., 2009). Some of the youth-serving CBOs we study may thus have a lasting impact on well-being and cultures of health among those who face a disproportionate risk of poor health outcomes.

### 3.1 Data and methods

Data come from Terriquez's ongoing research on youth civic engagement in thirteen low-income urban and semi-rural California communities involved in the Building Healthy Communities (BHC) Initiative. As part of BHC, these organizations received some grant funding from the California Endowment (See: http://www.calendow.org/building-healthy-communities/). The Endowment financed a portion of the research presented here, as did grants from the Bill and Melinda Gates Foundation, the Spencer Foundation, Atlantic Philanthropies, and the Charles Stewart Mott Foundation. We evaluate survey, semi-structured interview, and documentary data to demonstrate ways that CBOs can empower individuals, foster solidarity, and facilitate civic action around health-related concerns. Institutional Review Board approval for the survey and in-depth interviews was given by the Office for the Protection of Research Subjects at the University of Southern California. Our findings are illustrative; further research is needed to better understand the conditions that enable CBOs to promote cultures of health and to examine variations in the extent to which CBOs impact well-being at the individual and community levels.

We begin by outlining how CBOs can empower individual participants and provide health-related benefits. To this end, we rely on data from in-person paper surveys that were administered to youth involved in one of 68 out of the 69 non-profit youth-serving CBOs that received funding as part of the BHC Initiative in 2013–2015. The CBOs focus on leadership development and involve members in activities that range from growing community gardens and mediating peer conflicts, to learning about participants' cultures, health issues, or community problems. The majority of CBOs also provide academic or college preparatory guidance, and a minority offer art or athletic activities. Twenty-one groups engage members in advocacy or grassroots organizing about school and neighborhood concerns. The target population for the survey was “core youth members”, that is, young people who regularly participated in the CBOs. The modal group membership size was 15–30 individuals, but some organizations had fewer than 10 youth members, and a handful had over 50. A total of 1210 youth, ages 18–26, completed the survey, with a 91% response rate.

Study participants were asked questions about their demographic background and involvement in the organization. They were also asked to indicate the degree to which they benefited from their CBO involvement. With a couple of exceptions, questions did not ask directly about health benefits, and instead asked how the CBO facilitated their civic development. As explained above, the acquisition of civic skills and development of political efficacy can improve psychological empowerment, mental health, and health-related behaviors (Christensen and Person, 2012, Shinn and Toohey, 2003). Youth were also asked about educational and career-related outcomes that can have indirect effects on health.

To explore how CBOs can promote solidarity, we draw on a follow-up telephone survey targeting all of the above CBO participants who were still in high school and whose contact information remained current at the time of the follow-up. The resulting survey sample includes 440 teens. This subsample was asked, among other things, two questions related to solidarity and health: one question asked to what degree the respondent feels connected to others working to improve society; the second asked to what degree the respondent cares deeply about health issues.

Selection issues are a concern. Individuals engaged in CBOs are not representative of the larger youth population. We take a step to address self-selection by comparing the teen sample data to survey data from a representative sample of California adolescents, ages 13–17, who participated in the California Health Interview Survey (CHIS) in 2013–14 and who were asked many of the same questions as CBO participants. CHIS participants were sampled at the household-level through random digit dialing of landline and cell phone numbers. When present, one adolescent per household was surveyed; in households with two or more adolescents, one was randomly selected. Weighted results from the representative CHIS sample contextualize the CBO sample, and provide a tentative assessment as to whether CBO membership is positively associated with solidarity around health concerns.

We further flesh out the role of CBOs in promoting solidarity by analyzing semi-structured, in-person interview data from 85 current and former CBO members. Youth were interviewed at all 13 BHC sites, although 65 interviews come from just 6 sites and were collected in 2011–12 as part of the California Young Adult Study (Terriquez, 2015). As with the surveys, youth respondents were asked for informed consent; consent was also obtained from parents for those under age 18. Interviewees were selected using purposive, nonprobability quota sampling based on their race/ethnicity, gender, and other demographic characteristics. Interviewees were asked a range of questions about their family background, schooling experiences, civic participation, and other topics related to the transition to adulthood. We inductively coded respondents' discussions of their experiences in BHC-affiliated civic groups from fully transcribed interviews. We report on emergent themes, with a focus on solidarity. Respondents were not explicitly asked about the development of solidarity in their group; rather, this theme emerged from the coding process.

Finally, to understand how CBOs can promote community well-being, we use data from the semi-structured interviews with youth, along with interviews with at least two adult staff or community leaders from each of the 13 BHC sites, and archival data (organizational documents and media reports). All CBOs in this study engaged their young members in civic activities aimed at making community improvements with varying levels of success and intensity. CBOs sought to engage youth in promoting community well-being (in general terms) by addressing violence prevention, health care access, educational equity, economic opportunity, and so forth. For illustrative purposes, we select three distinct cases in which CBOs facilitated policy changes that had clear and varying implications for community health. We do not claim that all CBOs have the same level of impact, but rather we highlight cases that demonstrate what is possible, not necessarily typical.

### 3.2 Survey sample descriptions

Table 1 describes the three survey samples used for analysis. The first column reports descriptive statistics for the entire sample of CBO youth participants, aged 13–26, and the second column for the subsample of high school CBO members (hereafter the “CBO adolescent sample”). The third column provides data on the CHIS comparison group, representative of California's adolescent population. We include basic demographic and socio-economic characteristics, indicators of academic success (for those in high school) and data about the activities in which members of the CBO samples participated.

As shown in Table 1, 69% of respondents in the full CBO sample come from immigrant families. Almost all are racial minorities: 62% are Latino, 21% are African Americans, and 13% are Asian Pacific Islanders (mostly of Southeast Asian refugee backgrounds). Given participating CBOs' location in high-poverty communities, group members are overwhelmingly from socio-economically disadvantaged family backgrounds: 79% report being low-income (as measured by free and reduced lunch eligibility), and only 10% are being raised by parent(s) who hold a four-year college degree. Women outnumber men; the average age is 17.

The CBO adolescent sample, relative to the full CBO sample, has a slightly higher proportion of young people from immigrant, Latino, and Asian backgrounds, and a greater over-representation of women. Adolescent respondents tend to be performing well in school—66% report receiving mostly Bs or higher grades—but a small minority (11%) have mostly C grades or lower.

The racial/ethnic minority and low-income profile of the CBO adolescent sample does not reflect California's overall adolescent population. CHIS data indicate that a smaller proportion of California's adolescents come from immigrant, racial minority, and low-income backgrounds. Thus the youth in the CBO sample reflect low-income racial minority groups especially vulnerable to negative health outcomes (Dowd et al., 2014, Mehta et al., 2013, Mulye et al., 2009). The CHIS sample is, on average, younger than both CBO samples, but the academic performance of the CBO adolescents is similar to that of the CHIS random sample.

## 4 Findings

We first provide descriptive statistics from self-reports on how CBO involvement benefited individual participants. We then draw on survey and semi-structured interview data to suggest that CBOs help build solidarities around health issues. Finally, we demonstrate how CBOs have the potential to influence policy to improve community health.

### 4.1 CBO participation and individual-level empowerment

Table 1 provides suggestive evidence that CBOs can empower individuals and develop their skills. Respondents in the two CBO samples were asked whether, through their organization, they had participated in a set of activities. Over half of all CBO participants reported joining in activities that prepared them to enroll or succeed in college. A significant proportion exercised leadership in their groups: 41% made important decisions, 39% made a public presentation, and 34% planned a meeting or event. Nearly a quarter (24%) canvassed or collected signatures, indicating involvement in some kind of campaign, while 15% wrote about a community issue. Some also engaged in exercise (27%) or the arts (23%). Responses from the adolescent CBO sample were somewhat similar, except that a higher proportion participated in college prep/academic success activities.

It is possible that the CBO youth began developing civic and leadership skills prior to joining their organization. All respondents were consequently asked to rate how their CBO involvement impacted their personal development—did it have *no impact*, *very little impact*, *some impact*, or *a lot of impact*? Such self-reports are, of course, subjective and might suffer from social desirability bias, although the range of responses indicates that participants were not reluctant about disagreeing, when appropriate. To be conservative, Table 2 only counts young people who reported “a lot” of impact.

Results indicate that CBO involvement had an impact on the self-reported civic capacity of a significant proportion of members, ranging from an improved ability to plan events and activities (43%), to improved ability to communicate with others (61%). Over half of respondents (56%) reported that their understanding of how government decisions impact the community had developed “a lot.” Youth also reported gains in personal empowerment and efficacy, with 47% saying that they learned “a lot” about their culture or ethnic group and two-thirds (66%) said that CBO involvement had “a lot of impact” on their ability to stand up for their beliefs.

The CBO survey asked a few direct items on young people's health. Three out of five respondents (60%) reported having learned “a lot” about health issues that impact the community, although only a bit over a third (36%) felt that CBO involvement led them to take better care of their personal health. Young people tend to be relatively healthy, perhaps in part explaining these responses. CBO effects on personal health might also not be direct or immediate. To the extent that higher levels of education are known to correlate with better health, it is worth underscoring that 42% of respondents reported learning a lot about college or career options, although less than a third (31%) felt participation improved their school grades a lot, perhaps reflecting ceiling effects given respondents' strong grades.

### 4.2 CBO participation and solidarities around health issues

CBOs can also develop and sustain cultures of engagement and health by building social networks, fostering solidarity and collective efficacy, and by promoting a shared commitment to collective well-being. Almost two-thirds of respondents (62%) in the adolescent CBO survey said they were in strong agreement with the statement that they felt connected to others working to improve society, compared to only 30% in the state-wide CHIS youth sample (see Table 3). Furthermore, 73% of adolescent CBO participants said that they care deeply about community health issues, compared to 50% in the CHIS sample. Since youth in the CBO sample chose to participate in their community organization prior to the survey, it is hard to know the extent to which CBO involvement had an effect independent of members' pre-existing attitudes, sense of solidarity, or interest in health, but survey responses are suggestive of influence. At a minimum, CBOs can likely contribute to solidarities around health by increasing social networks among young people who care about community health issues.

To complement survey data, we draw on semi-structured interview data collected from current and former CBO participants for insights into *how* CBOs foster solidarity. In line with prior research, we find that some CBOs help members develop a shared political analysis and understanding of community concerns (Ginwright and Cammarota, 2007, Terriquez, 2015). This process can be politicizing and motivate young people to work with others who share similar backgrounds. Darryl's experience is typical. A son of Filipino immigrants, he participated in several advocacy campaigns, including a CBO effort to reduce youth violence, by speaking out for increased funding for after-school programs in Oakland. “We learn things that we don't learn from the school,” Darryl explained. “We have more awareness of what's happening. If you're involved, you start watching documentaries, reading these articles, and that's when you start realizing that we need to fight for positive change.” Similarly, Felipe, a Mexican American deeply involved in a South Los Angeles organization, said, “A lot of the things we were learning about and working on were directly relevant to us—poverty, joblessness, violence, and crime. To be part of a collective and to be able to join with other people who want to change those things, I think, was motivating for a lot of us.”

Some CBOs create a venue to develop solidarity across race and immigrant background. CBOs accomplish this in part by engaging young people in “issues that affect everybody,” as one youth put it. In some CBOs, ethnic studies training educates members about the histories of economic and/or racial marginalization experienced by different groups in the organization. Such dialogues facilitate bonds that may not otherwise exist. Ignacia, a Long Beach resident and daughter of Mexican immigrants, explained, “In the high schools, there's always racial tensions. Here they taught us not to discriminate against people, so we actually get to know them.” To help develop solidarity with African American peers, her organization held workshops about the history of African American lynching and the “N” word. “Learning about all this racial stuff makes you more aware of the racial injustice, and other changes that needed to be made,” claimed Ignacia, who believed that her organization fostered collaboration and strong friendships across racial lines.

Not all CBOs in this study are located in racially diverse neighborhoods or seek to reflect the ethno-racial composition of their communities, but 25 out of 68 (37%) CBOs contained significant representation (25% or more) of two or more racial/ethnic groups. Cross-racial solidarity can subsequently emerge, as articulated by eighteen-year-old Andrea, a member of a South Los Angeles organization: “We all have the same struggle. We're all people of color, and our circumstances are the same regardless of our skin color or our cultural backgrounds.” Having worked with her African American and Latino peers to learn about and tackle violence, incarceration, and low college-going rates among youth in her neighborhood, Andrea, like many others, believed that her CBO was “fostering that safe space where we could come together and create positive changes.”

Beyond intra-organizational solidarity, CBOs also enabled members to develop ties with others outside the organization. This occurred when CBOs worked to develop a shared understanding of the structural causes of local problems and engaged members in grassroots organizing and advocacy. Lucy, a 16-year-old member of a Cambodian youth organization, spoke with excitement about how CBO participation enables her to form relationships and solidarity with peers across Long Beach:

Through my involvement, I got to work with many different people, and other clubs. We support each other on many of the campaigns that affect our schools' policies and our own reproductive health. I think it's important to work with them because without our allies, our movement is not as strong.

CBO efforts enabled Lucy and her Cambodian peers to attend meetings, strategize, and personally connect with Latino and African American youth outside their organization, as well as with adult allies. In doing so, CBOs can orient members to inter-ethnic and inter-racial ties at a formative stage in their lives. As Sun, an Oakland resident and son of a refugee from Laos, put it: “I learned to work in coalition with other folks who aren't necessarily from the same background. I learned how to connect with folks personally, but also see how issues affect not only my ethnic community, but different communities, and be able to work together.” Such solidarity and the ties undergirding it are, we posit, vital for fostering a culture of engagement and health, and the collective action often necessary to mobilize for change in low-income, immigrant, and minority communities.

### 4.3 CBOs' impact on community well-being

Most CBOs also facilitated young people's involvement in civic activities and campaigns aimed at improving *community* well-being, broadly defined, not just individual skills, empowerment or feelings of solidarity. We draw on semi-structured interview data and documentary material to offer three examples of how youth, through their CBO involvement, sought to contribute to community health by influencing policy and programing. The three cases are not necessarily typical, but illustrate distinctive strategies and targets for structural changes.

Our first example comes from the semi-rural region of South Kern County located outside Bakersfield, California. In this community the Dolores Huerta Foundation and Boys and Girls Club involved youth members, many who are the children of Mexican immigrant farmworkers, in advocating for cleaner, safer parks. Youth were concerned that local parks were neglected, full of trash, and did not offer an inviting environment for exercise and recreation. Initially, youth painted over graffiti and picked up beer caps, broken glass bottles, and cigarette butts. But while such volunteer efforts are often celebrated in accounts of civic engagement (Putnam, 2000), they often only make limited, short-term changes that do not address systemic environmental or structural problems (Eliasoph, 2013). Realizing that volunteer efforts were not enough to keep parks clean and safe, youth and adult allies initiated the “Beautiful Parks, Healthy Communities” campaign to pass and enforce ordinances to prevent alcohol and tobacco consumption in public parks. Youth canvassed neighborhoods, participated in and made presentations at community and government meetings, and held one-on-one meetings with decision-makers to build support among residents, other youth, Parks and Recreation Commissioners, and the County Board of Supervisors.

In early 2014, their efforts were rewarded: the Kern County Board of Supervisors voted to restrict the use of alcohol and tobacco in local parks. Soon after, park rangers began educating community members about the ordinances and within weeks, the Sheriff's department began issuing citations for violations. According to Bob Lerude, the Kern County Director of Parks and Recreation, the ordinances have created a more inviting park environment, enabling local residents to take better advantage of this community health resource. Moreover, Lerude notes that young people and their allies continue to participate in park improvement efforts. He credits the local CBOs with developing young people's leadership to shape park programming and policies, explaining: “I'm in charge of parks all over Kern County, and I don't see youth involvement like this anywhere [else]. Youth aren't just talking about what they want; they are helping make it happen. We really don't have many resources, and the community's involvement really makes a difference” (Interview with Bob Lerude, 5/1/2015).

Our second example comes from the low-income City of Santa Ana, Orange County, where young people from various organizations built a coalition to improve school discipline and safety, and shaped the school district's budget priorities. Members of the Youth Empowerment Network, Orange County DREAM Team, and the Boys and Young Men of Color Initiative sought to change punitive school discipline policies that resulted in high rates of suspensions for minor offenses (such as talking back to the teacher) and frequent involvement of police in more serious allegations, which sometimes put undocumented students at risk of deportation. Youth and their allies advocated for alternative restorative justice policies that repair harms caused by offenses and address the underlying causes of violence. In a related effort, youth members of the LGBT Center OC sought to address bullying and create a safer school climate for LGTBQ youth in ways that aligned with the principles of restorative justice.

Presenting a united front, the youth groups participated in public hearings, attended planning meetings, met with school district officials, and mobilized peers to urge the district to dedicate resources to these policies in the Local Control Accountability Plan. In response, Santa Ana Unified School District (SAUSD) allocated $7 million to promote a safer school climate through restorative programming and by offering “Safe and Sensitive School” and “Anti-Bullying Awareness” trainings to address harassment of LGBTQ and other marginalized youth (Gerda, 2014). Unfortunately, a year later, SAUSD had taken only limited steps to implement their commitments. This case underscores the importance of organizational homes for community-based health initiatives, to sustain action and accountability over time, and why attention to ‘meso-level’ groups needs to be a part of a broad health and wellness framework.

Nevertheless, according to Laura Kanter, Director of Policy, Advocacy, and Youth Programs for the LGBT Center OC, the youth and community-led effort resulted in a “cultural shift, where the District staff had to start getting used to hearing from the community.” As part of an ongoing effort to improve school safety and school climate, youth, along with parents and other allies, are participating alongside district officials in a School Climate Committee to press the district to implement community demands, evidence of CBOs' role in giving community members a long-term voice in institutional and cultural shifts.

In a third example, in Fresno County, Barrios Unidos, the Fresno Immigrant Youth Alliance, and other youth-serving organizations joined a successful intergenerational grassroots effort to pressure the County Board of Supervisors to re-fund the suspended Medically Indigent Services Program. The program, which provides specialty medical care for undocumented immigrants and the poor, was a source of controversy, in part because most California counties do not fund services for undocumented immigrants not covered by the Affordable Care Act (Dembosky, 2014). Young people educated their peers and broader community on the issue, mobilized community members to attend public meetings, and met with elected officials and other government representatives. Adult allies were prominent in this campaign, but youth testimony was critical at key public meetings and hearings. Many youth were involved because they were undocumented, or had parents or other family members who had little to no access to health insurance. The campaign was a success: County Supervisors voted 3–2 to spend $5.6 million for health services by deferring repayment of road funds to the state. Inspired by the victory, youth in Fresno remain active in local and statewide “Health4All” campaigns to expand medical insurance to undocumented immigrants. Collective action facilitated by CBOs helped shift, if only slightly, the structural challenges faced by undocumented residents in Fresno County.

## 5 Concluding discussion

We argue that civic infrastructures of formalized community-based organizations can play an important role in generating, diffusing and maintaining a culture of health and engagement that benefits marginalized populations most at risk for poor health outcomes. Extant scholarship already underscores the importance of “meso-level” factors, notably social networks and social identities, for health and well-being (Berkman et al., 2014, Cruwys et al., 2013, Giordano and Lindstrom, 2010, Smith and Christakis, 2008, Jetten et al., 2012). These effects stand distinct from individual and structural determinants of health. We contribute to conceptualizing this “meso-level” of analysis by drawing attention to the role of community-based organizations for individual and community health and well-being.

CBOs can produce individual-level health benefits by fostering civic engagement, psychological efficacy, social relations, a sense of solidarity and collective identity. We make the further claim that attention to somewhat formalized groups – ones that have some history, collective identity, and ongoing social relations in common spaces over time – also elucidates how individual action can produce change in the structural determinants of health. To the extent that CBOs and other groups mobilize people to have a voice in health-related policy and programming, civic infrastructures carry benefits for communities. While research highlights the benefits of collective action for improving individual mental health, reducing violence and increasing well-being (Sampson et al., 1997, Shinn and Toohey, 2003, Van Zomeren et al., 2008), less attention has been paid to how collective mobilization for structural change increases community health (but see Minkler and Wallerstein, 2008). We believe that the “culture of health” perspective being advanced by the Robert Wood Johnson Foundation not only invites, but requires an approach attentive to CBOs and cultures of engagement. Such an approach is also directly germane to the health challenges facing California, which is home to one of the most diverse populations in the United States and characterized by very high income inequality, from the riches of tech professionals in Silicon Valley to the poverty of farm workers in the Central Valley.

In making our argument, we do not want to suggest that CBOs should take the place of government and health care institutions. Indeed, CBO-led engagement likely works best in environments that promote partnership between public and civic institutions (Bloemraad, 2006, Hertzman and Siddiqi, 2013). Nor do we think a simple-minded call for more volunteering or civic engagement will erase structural inequalities (see also Eliasoph, 2013). Rather, CBOs can function as a vehicle for engaging marginalized populations in sustained individual-level and community-level change.

We illustrated the potential for this approach by looking at the self-reported effects of young Californians' participation in CBOs. Given the nature of our data, which includes nonprobability sampling of organizations in low-income, minority communities and selection effects as to which youth join and stay in such organizations, we need more systematic data to pinpoint the health effects of CBO presence and involvement. Even so, youth in our study report that their engagement improves communication skills, political and health knowledge, and their ability to organize collectively; there is also evidence of a heightened sense of connection to others and interest in health issues. Our illustrative examples show how CBO-sponsored campaigns may improve community health and well-being by ensuring safe spaces to play, pushing for health services at schools, advancing restorative justice approaches to tackle school discipline and violence, and securing funding for health services for undocumented immigrants. Future studies should employ a longitudinal data collection strategy to gather data from both CBO participants and non-participating community members to measure individual and community health and well-being effects more precisely over time. Future research should also investigate the organizational structures and social conditions that enable CBOs to most effectively engage members and produce positive health outcomes (see also Jetten et al., 2014).

In short, if a “culture of health” means the promotion of individual and community well-being, creating and maintaining healthy physical and social environments, and supporting equitable well-being for everyone, then an important way to promote these goals is to facilitate a “culture of engagement.” Civic infrastructures are a critical foundation to cultures of engagement, and thus to positive cultures of health.

## Figures and Tables

**Table 1:** Youth samples, descriptive statistics.

	CBO full sample (N = 1210)	CBO adolescent sample (N = 440)	CHIS sample (N = 786)^a^
Immigrant family	69%	72%	55%
*Race/ethnicity*			
Latino	62%	65%	46%
White	1%	<1%	31%
Black	21%	18%	7%
Asian-Pacific Islander	13%	15%	14%
Other	2%	2%	3%
*Socioeconomic background*			
Raised by parent(s) with Bachelor's degree	10%	9%	41%
Low-income	79%	93%	44%
*Gender*			
Male	45%	38%	53%
Female	55%	62%	47%
*Average age*	17.3	16.2	15.5
*Self-reported grade point average (GPA)*			
Mostly Ds or lower	NA	2%	3%
Mostly Cs and Ds	NA	5%	3%
Mostly Cs	NA	4%	5%
Mostly Bs and Cs	NA	22%	18%
Mostly Bs	NA	10%	12%
Mostly As and Bs	NA	38%	39%
Mostly As	NA	18%	20%
**How young people have been involved in their group**			
College prep/success activities	52%	62%	NA
Made important decisions	41%	40%	NA
Made a public presentation	39%	40%	NA
Planned a meeting or event	34%	31%	NA
Physical exercise at least once a week	27%	27%	NA
Performed or showcased art	23%	21%	NA
Collected signatures/canvassing	24%	29%	NA
Wrote about community issue	15%	14%	NA
Facilitated restorative justice circle	14%	13%	NA
None of the above	12%	10%	NA

Results may not add up to 100% because of rounding.

a Weighted results.

**Table 2:** Youth reports on CBO involvement and healthy development (N = 1210).

The degree to which youth feel that their involvement in their CBO has impacted their development	
*Percent who responded “A lot”*	
**Civic skills**	
Improved ability to communicate with others	61%
Developed better understanding of how government decisions impact community	56%
Improved ability to speak in public	48%
Improved ability to plan events and activities	43%
**Empowerment and efficacy**	
Learned to stand up for beliefs	66%
Learned about health issues that impact the community	60%
Learned more about own culture or ethnic/racial group	47%
**Health and education outcomes**	
Learned about college or career options	42%
Taken better care of personal health	36%
Improved school grades	31%

**Table 3:** Indicators of solidarity around health issues, CBO and California youth.

	CBO adolescent sample (N = 440)	CHIS sample^a^ (N = 786)
Percent indicating “strong agreement”		
Connected to others working to improve society	62%	30%
Cares deeply about community health issues	73%	50%

a Weighted results.
